# Supplementary material for: New and Effective Inhibitor of Class I HDACs, Eimbinostat, Reduces the Growth of Hematologic Cancer Cells and Triggers Apoptosis
Source: Pharmaceutics. 2025 Mar 25;17(4):416. doi: 10.3390/pharmaceutics17040416 (PMC12030756; doi:10.3390/pharmaceutics17040416)
Supplement: Supplementary file 1 [file pharmaceutics-17-00416-s001.zip › pharmaceutics-3518047-supplementary.pdf]

Supplementary materials

Interpretation of ^{1}H NMR and ^{13}C NMR spectra of Eimbinostat

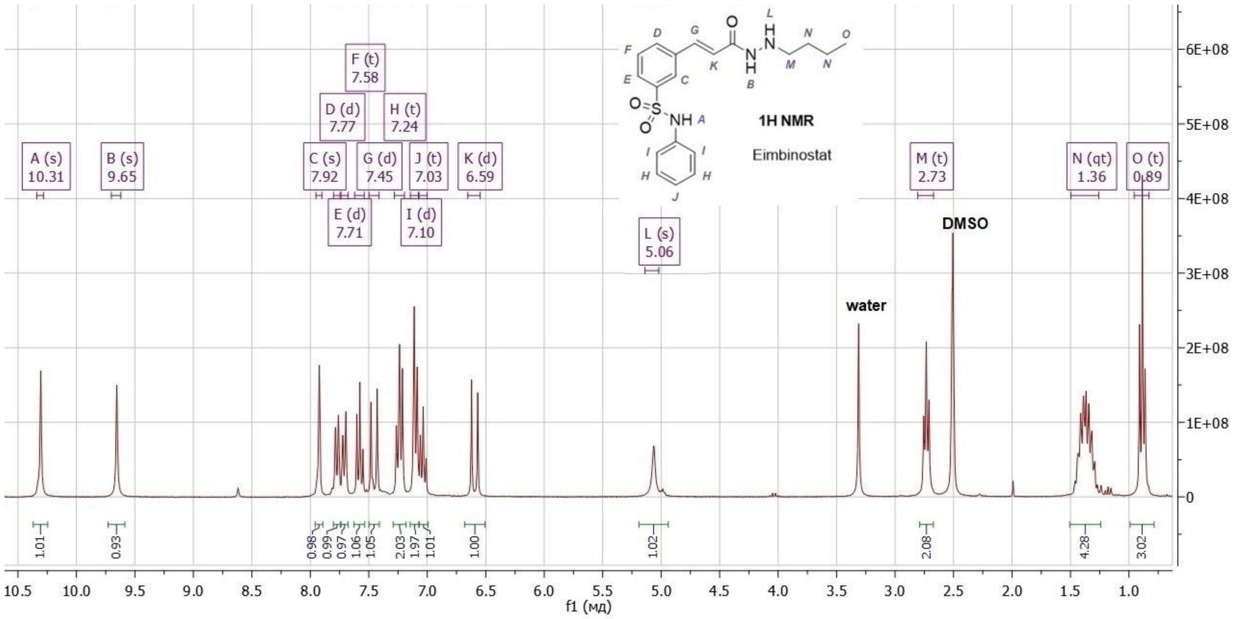

Figure S1. ^{1}H NMR (300 MHz, DMSO) δ 10.31 (s, 1H), 9.65 (s, 1H), 7.92 (s, 1H), 7.77 (d, J = 7.7 Hz, 1H), 7.71 (d, J = 8.1 Hz, 1H), 7.58 (t, J = 7.8 Hz, 1H), 7.45 (d, J = 15.9 Hz, 1H), 7.24 (t, J = 7.8 Hz, 2H), 7.10 (d, J = 7.5 Hz, 2H), 7.03 (t, J = 7.3 Hz, 1H), 6.59 (d, J = 15.8 Hz, 1H), 5.06 (s, 1H), 2.73 (t, J = 6.8 Hz, 2H), 1.36 (qt, J = 13.8, 6.7 Hz, 5H), 0.89 (t, J = 7.1 Hz, 3H).

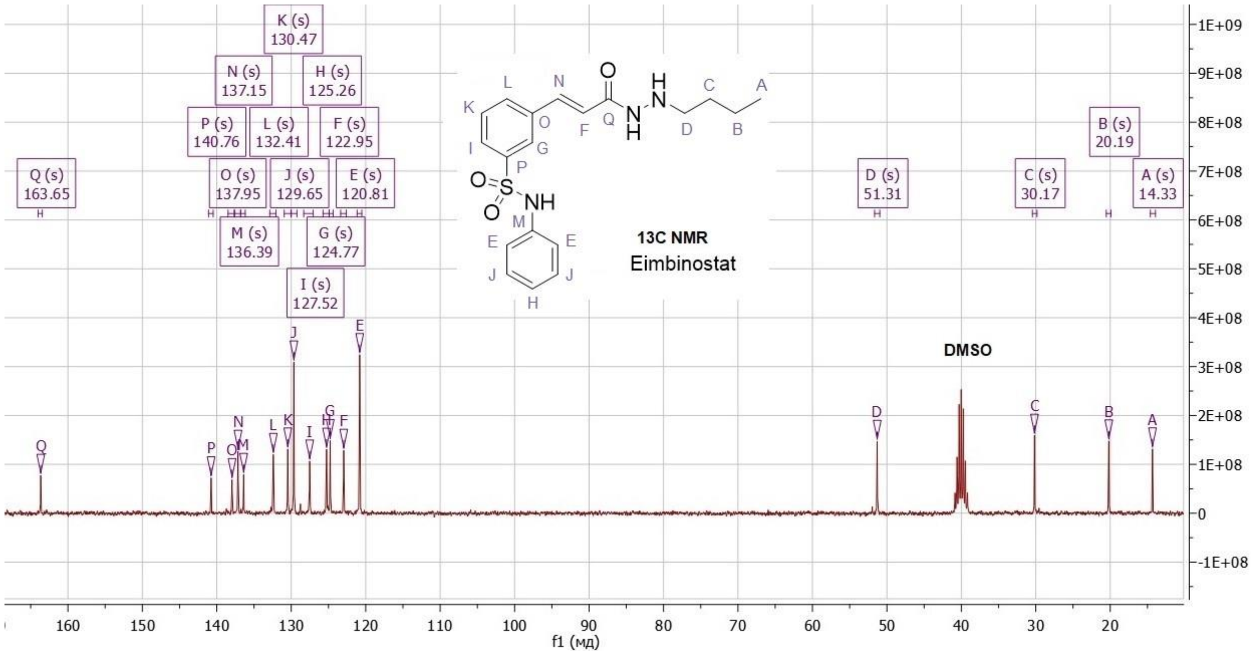

Figure S2. ^{13}C NMR (75 MHz, DMSO) δ 163.65 (s), 140.76 (s), 137.95 (s), 137.15 (s), 136.39 (s), 132.41 (s), 130.47 (s), 129.65 (s), 127.52 (s), 125.26 (s), 124.77 (s), 122.95 (s), 120.81 (s), 51.31 (s), 30.17 (s), 20.19 (s), 14.33 (s).

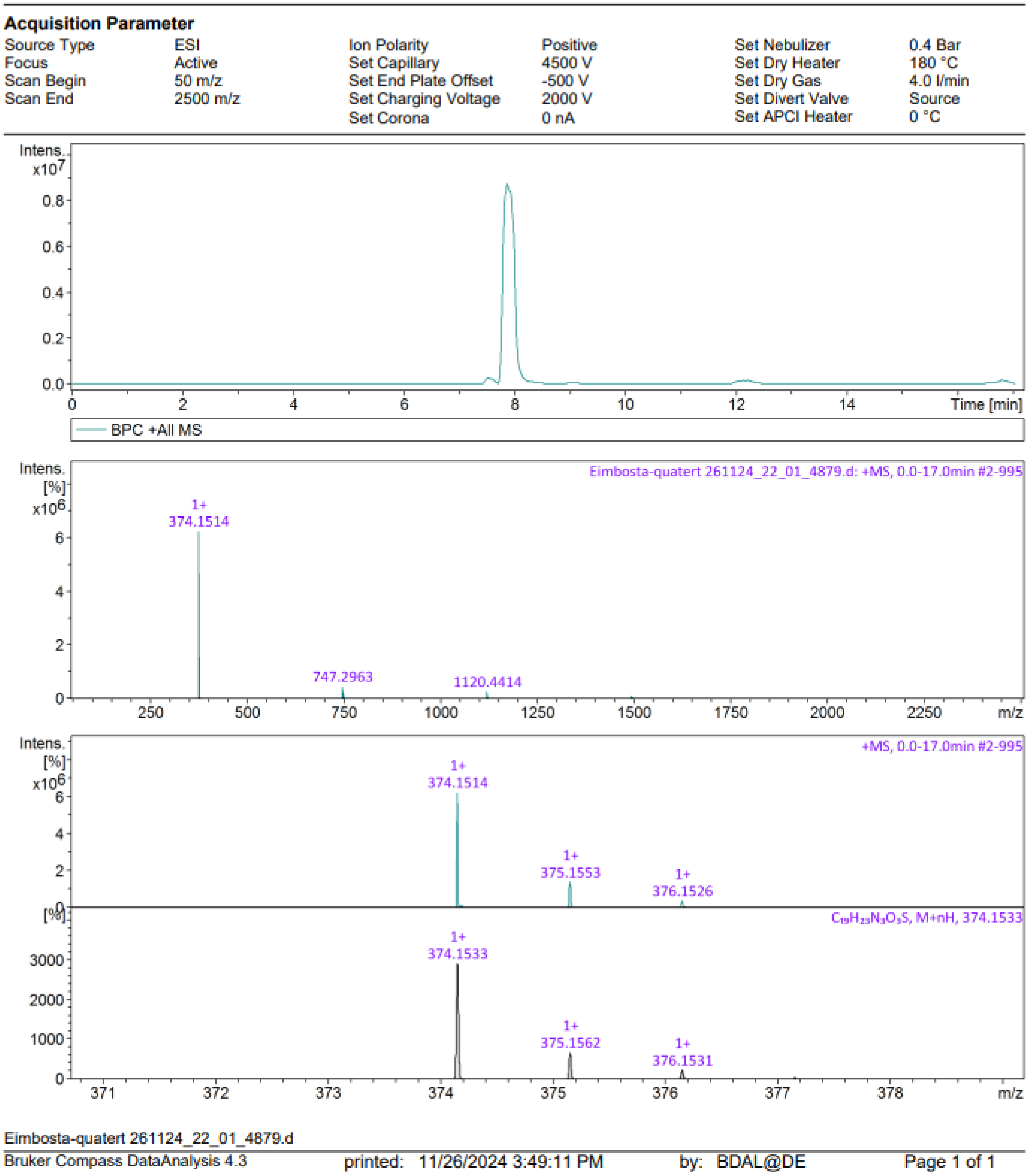

Figure S3. HRMS spectrum of Eimbinostat (1)

Molecular ion [M+H]+ (m/z, z=1):

High-resolution mass spectra were recorded on a micrOTOF-Q II device (Bruker Daltonics, Bremen, Germany) by electrospray ionization mass spectrometry (ESI-MS). Measurements were carried out in positive ion mode; samples were injected into the mass-spectrometer chamber from an HPLC system Agilent 1260 (Agilent Technologies Inc., Santa Clara, CA, USA). The following parameters were used: capillary voltage 4500 V; mass scanning range: for positive ion polarity m/z 50–2500; external calibration with LC/MS Calibration standard for ESI-TOF (Agilent Technologies Inc., Santa Clara, CA, USA); gas pressure 0.4 bar; nitrogen spray gas (4 L/min); interface temperature: 180 °C; flow rate 200 μL/min. Molecular ions in the spectra were analyzed and matched with the appropriately calculated m/z and isotopic profiles in the Bruker Data Analysis 4.0 program. Dry sample (1) were dissolved in 100% acetonitrile.

Injected into the mass-spectrometer spray chamber from an Agilent 1260 HPLC chromatograph equipped with an Agilent Poroshell 120 EC-C18 column (3.0 × 50 mm; 2.7 μm) (Agilent Technologies Inc., Santa Clara, CA, USA) and a compatible pre-column cartridge using an autosampler. The column was eluted with a mixture of Formic acid (A) and acetonitrile (B) in a gradient concentration with a flow rate of 200 μL/min in the following gradient parameters: 17% B for 2 min, 17–100% B for 5 min, 100% B for 5 min, 100–17 % B for 2 min, and 17 % B for 2 min.

The retention time on HPLC (System I) was 7.9 min. HRMS (ESI) of $C_{19}H_{23}N_3O_3S$: positive mode m/z calcd. for $[M+H]^+$ 374.1533, found: 374.1515, calcd. for $[2M+H]^+$ 747.2993, found: 747.2963, calcd. for $[3M+H]^+$ 1120.4453, found: 1120.4414.

Table S1. Sequences of primers used in study.

	**forward**	**reverse**
GAPDH	GAGCCCGCAGCCTCCCGCT	GCGCCCAATACGACCAAATC
HDAC1	TGCTAAAGTATCACCAGAGGGT	GGAGCGGGTAGTTAACAGCA
HDAC2	ATGGCGTACAGTCAAGGAGG	TGCGGATTCTATGAGGCTTCA
HDAC3	CCAGAGAGTCAGCTCCACA	CGGAAATTACTTCTTCCCTGGCA
HDAC4	GGCCCACCGGAATCTGAAC	GAACTCTGGTCAAGGGAACTG
HDAC5	TCTTGTCGAAGTCAAAGGAGC	GAGGGGAACTCTGGTCCAAAG
HDAC6	AAGAAGACCTAATCGTGGGACT	GCTGTGAACCAACATCAGCTC
HDAC7	GGCGGCCCTAGAAAGAACAG	CTTGGGCTTATAGCGCAGCTT
HDAC8	TCGCTGGTCCCGGTTTATATC	TACTGGCCCGTTTGGGGAT
HDAC9	AGTAGAGAGGCATCGCAGAGA	GGAGTGTCTTTCGTTGCTGAT
HDAC10	CAGTTCGACGCCATCTACTTC	CAAGCCCATTTTGCACAGCTC
HDAC11	ACCCAGACAGGAGGAACCATA	TGATGTCCGCATAGGCACAG
Bcl2	TGAACTGGGGGAGGATTGTG	CGTACAGTTCCACAAAGGCA
BCL2L1	GCGTGGAAAGCGTAGACAAG	GTCAGGAACCAGCGGTTGAA
TP53	ACCTATGGAAACTACTTCCTGAAA	CTGGCATTCTGGGAGCTTCA
NF-kB	ACCCGGCTTCAGAATGGCA	GGTATGGGCCATCTGCTGTT
CCNB1	CCTCTCCAAGCCCAATGGAA	TGGTCTGACTGCTTGCTCTT
CCND2	GTTCCTGGCCTCCAAACTCA	CTTGATGGAGTTGTCGGTGTAAAT
E2F1	TGACCCAGGACCTCCGACAG	GCCTTGTTTGCTCTTAAGGGAGAT
CDKN1A	ACATCGCCAAGGAAAAACGE	GTCTGTTTCGGTACTGTCATCC
CDKN1B	GGCTAACTCTGAGGACACGC	TGGGGAACCGTCTGAAACAT
CCNA1	GCCTCCTGTCTGGTGGGA	TGCAGTGCATTGCTTCAGAC

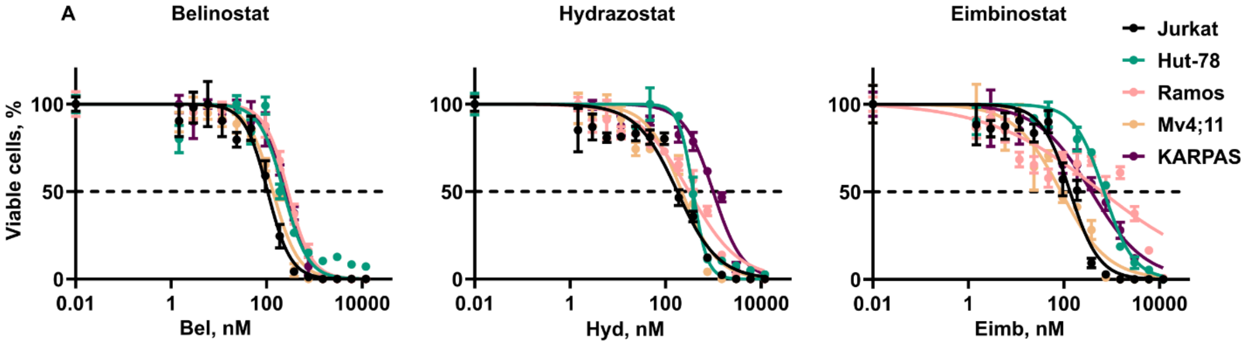

B

	Jurkat	Hut-78	Ramos	MV4;11	KARPAS
Bel	110.0 nM	252.3 nM	280.9 nM	135.6 nM	251.1nM
Hyd	175.0 nM	380.4 nM	308.7 nM	217.9 nM	1180.0 nM
Eimb	134.3 nM	686.8 nM	544.4 nM	86.79 nM	374.1 nM

log2(relative ic50): 1, 0, -1

Figure S4. The cytotoxicity of Belinostat, Hydrazostat, and Eimbinostat. A) Dose-effect curves represent the viability of cells after 72h exposition of cells with the inhibitors. B) Heat map table represents Half-maximal inhibitory concentrations (IC50) of studied compounds.
